# Pineal Gland Calcification in Kurdistan: A Cross-Sectional Study of 480 Roentgenograms

**DOI:** 10.1371/journal.pone.0159239

**Published:** 2016-07-14

**Authors:** Kahee A. Mohammed, Eric Adjei Boakye, Honer A. Ismail, Christian J. Geneus, Betelihem B. Tobo, Paula M. Buchanan, Alan P. Zelicoff

**Affiliations:** 1 Department of Epidemiology, College for Public Health and Social Justice, Saint Louis University, Saint Louis, Missouri, United States of America; 2 Center for Outcomes Research (SLUCOR), Saint Louis University, Saint Louis, Missouri, United States of America; 3 Department of Radiology, School of Medicine, University of Duhok, Duhok, Kurdistan–Iraq; 4 Department of Environmental and Occupational Health, College for Public Health and Social Justice, Saint Louis University, Saint Louis, Missouri, United States of America; University of Brescia, ITALY

## Abstract

**Objective:**

The goal of this study was to compare the incidence of Pineal Gland Calcification (PGC) by age group and gender among the populations living in the Kurdistan Region-Iraq.

**Methods:**

This prospective study examined skull X-rays of 480 patients between the ages of 3 and 89 years who sought care at a large teaching public hospital in Duhok, Iraq from June 2014 to November 2014. Descriptive statistics and a binary logistic regression were used for analysis.

**Results:**

The overall incidence rate of PGC among the study population was 26.9% with the 51–60 age group and males having the highest incidence. PGC incidence increased after the first decade and remained steady until the age of 60. Thereafter the incidence began to decrease. Logistic regression analysis revealed that both age and gender significantly affected the risk of PGC. After adjusting for age, males were 1.94 (95% CI, 1.26–2.99) times more likely to have PGC compared to females. In addition, a one year increase in age increases the odds of developing PGC by 1.02 (95% CI, 1.01–1.03) units after controlling for the effects of gender.

**Conclusion:**

Our analysis demonstrated a close relationship between PGC and age and gender, supporting a link between the development of PGC and these factors. This study provides a basis for future researchers to further investigate the nature and mechanisms underlying pineal gland calcification.

## Introduction

The pineal gland is part of the epithalamus and is situated in the midline in the posterior part of the third ventricle [1]. The gland has two laminae and typically measures 7×6×3 mm in size [2]. The gland is responsible for production and secretion of melatonin, a neuroendocrine hormone well known for its effect on the modulation of sleep patterns in both circadian and seasonal rhythms [3,4]. Melatonin also has many other effects, including anti-inflammation, antioxidant, and neuroprotection [5–10].

The incidental finding of pineal gland calcification (PGC) has commonly been observed using conventional skull roentgenogram and computed tomography (CT) scan. There is great variability in the incidence of PGC in the general population in different countries and among different races. In general, the incidence is higher in western countries compared to Asian and African countries [11]. Reports indicate a significant difference in the incidence of PGC in White Americans (16%) and African Americans (9.8%) [12]. Various rates have been reported in some African and Asian countries, with the incidence being 1.3% in Gambians, 5% in Nigerians, 9.9% in Japanese, 15.6% in Fijians, and 19.2% in Indians [11,13–15]. Though calcification of the gland increases with age, it has been observed in children as young as 2 years old [16]. The incidence of PGC in different age groups among Caucasians was reported by Wurtman et al. to be 2% in those 3 to 12 years old, 46% in those 13 to 40 years old, and 69% in those above the age of 40, indicating an increase with age [17].

Decline in melatonin secretion is an age related process and has been linked to a variety of neurodegenerative diseases, such as Alzheimer disease, epilepsy, Parkinson disease, depression, stroke, and cancer[16,18–24]. Pineal gland has predilection for calcification and it has long been hypothesized by many authors that melatonin deficit may be caused by calcification of pineal gland [2]. Mahlberg et al. reported that patients with Alzheimer disease have significantly higher degree of pineal calcification than healthy elderly patients [25]. Similarly, PGC has been associated with schizophrenia, and multiple sclerosis [26,27].

Because abnormal pineal function is implicated in PGC, its role in several neurodegenerative disorders has received considerable attention in research. The global burden of neurodegenerative diseases, including Middle Eastern regions, has steadily grown as life expectancy rises and the older-age population increases. For example, in a recent study conducted in northern Israel, the prevalence of Alzheimer’s disease was approximately 10% and the rate of mild cognitive impairment was found to be approximately 32%, which is atypically high [28]. Another analysis in an urban population of Turkey found that the burden of Alzheimer’s disease (11%) and dementia (20%) are similar with those observed in the Western hemisphere [29]. Both Israel and Turkey exhibit socio-demographic, geographic, and climatological characteristics comparable to other surrounding regions, including Kurdistan. Due to its health implications, it is imperative to establish baseline evidence for burden of PGC in Kurdistan.

To our knowledge, no study has focused on the incidence of PGC and its relationship with age and gender in the Kurdistan Region-Iraq. The purpose of this present study was therefore to determine the overall incidence of PGC in Duhok, Kurdistan Region-Iraq, and to compare the incidence of PGC between different age categories and gender, using plain skull roentgenographic examinations. This study also aimed to investigate the association between age, gender, and PGC.

## Methods

### Study design, setting, and population

This prospective study involved examination of skull X-rays of 480 patients aged 3 to 89 years old (data in S1 Dataset_Original and S1 Dataset_Analytic), who were consecutively referred to the radio diagnostic center at a large teaching public hospital in Duhok, Iraq between June and November of 2014. The majority of patients in the city of Duhok and surrounding rural areas, who require either emergency care or are referred from primary healthcare centers, are seen in this main hospital. These patients were referred for skull X-ray examinations as part of routine neurological investigations for various clinical presentations. Indications included headache (27.1%), neck pain (22.1%), dizziness (12.9%), snoring (12.1%), trauma (10.6%), vertigo (9.6%), seizure (3.5%), psychological upset (1.0%), sinusitis (0.6%), numbness (0.2%), and tinnitus (0.2%). There were no exclusionary criteria for this study.

Verbal informed consent was obtained from all patients included in the study. For children (participants under the age of 18), verbal informed consent was obtained from their parents, caretakers, or guardians. Privacy procedures were explained to all patients, including assurance that no unique identifiers would be collected nor used for the study and all information would be kept confidential. No incentives were provided to study participants. Institutional Review Board (IRB) approval was granted for the conduct of this study and the verbal consent used from the University of Duhok School of Medicine, and the Duhok Governorate, Kurdistan Region-Iraq.

### Data collection

The presence or absence of pineal gland calcification was determined by observing the two standard views of the skull that were routinely obtained: Anteroposterior and Lateral film. The exposure factors employed in this study were 60–65 kVp for lateral films and 70–75 kVp for anteroposterior films, and 20 mAs for both. X-ray tube to screen focal distance was about 90 cm. Technically inadequate films were repeated with proper technique if possible or excluded from the study. All films were carefully reviewed by two radiologists searching for any calcified spicules in the region of pineal gland. If PGC was suspected in any of the views, pineal view was obtained as determined by Oon’s method [30]. However, we also considered and excluded extraneous calcification of other structures in the pineal area (i.e. in the falx cerebri, choroid plexus, and Habenular commissure) to help reduce misclassification error. The size of the calcified pineal gland was measured in millimeters (mm) using a caliper. Age, gender, size of the pineal gland, and the reason for requesting a skull X-ray were recorded for all patients.

### Measures

The dependent variable was calcification and was dichotomized as negative (to represent the absence of calcification in the pineal gland) and positive (to represent the presence of calcification in the pineal gland). The independent variables were age range and gender. Age was grouped into nine ranges: 10 years or younger, 11–20, 21–30, 31–40, 41–50, 51–60, 61–70, 71–80, and 81 years or older for the incidence analysis and utilized as a continuous variable for the logistic regression analysis.

### Statistical analysis

Bivariate logistic regression model were constructed to evaluate the association between PGC and age and PGC and gender. Crude Odds ratios (cORs) and 95% confidence intervals (CIs) were calculated. In a multivariable analysis, a logistic regression model was constructed to evaluate the association between PGC and age and gender. Adjusted Odds ratios (aORs) and 95% confidence intervals (CIs) were computed. Statistical significance was determined using a *p* ≤ 0.05 for all comparisons. Analyses were performed using SAS System for Windows Version 9.4 (SAS Institute Inc, Cary, North Carolina).

## Results

Table 1 summarizes descriptive statistics for the study population. Of the 480 individuals in the sample, 129 (26.9%) had a positive calcification diagnosis and 351 (73.1%) had a negative calcification diagnosis. Participants had a mean age of 37.6 years (*SD* = 21.8) and 57.7% were females. The average size of the calcified gland was 5.9 mm (*SD* = 1.3 mm) (Table 1).

**Table 1:** Demographic features and distribution of individuals by PGC and Incidence of PGC and its relationship with age and gender.

		Pineal gland calcification		Incidence of PGC %
	Total	Positive	Negative	
	mean ± *SD* or n(%)	mean ± *SD* or n (%)	mean ± *SD* or n (%)	
Overall	480 (100.00)	129 (26.88)	351 (73.12)	26.88
Age (in years)	37.64 ± 21.81	38.03 ± 11.43	37.53 ± 23.79	
Gender				
Female	277 (57.71)	64 (49.61)	213 (60.68)	23.10
Male	203 (42.29)	65 (50.39)	138 (39.32)	32.02
Age Category				
≤10	74 (15.42)	0 (0.00)	74 (21.08)	0.00
11–20	44 (9.17)	10 (7.75)	34 (9.69)	22.73
21–30	66 (13.75)	17 (13.18)	49 (13.96)	25.76
31–40	86 (17.92)	32 (24.81)	54 (15.38)	37.21
41–50	79 (16.46)	29 (22.48)	50 (14.25)	36.71
51–60	50 (10.42)	19 (14.73)	31 (8.83)	38.00
61–70	43 (8.96)	13 (10.08)	30 (8.55)	30.23
71–80	26 (5.42)	7 (5.43)	19 (5.41)	26.92
> 80	12 (2.50)	2 (1.55)	10 (2.85)	16.67
Size of the calcified gland (in mm)	5.91 ± 1.27			

**Fig 1:**
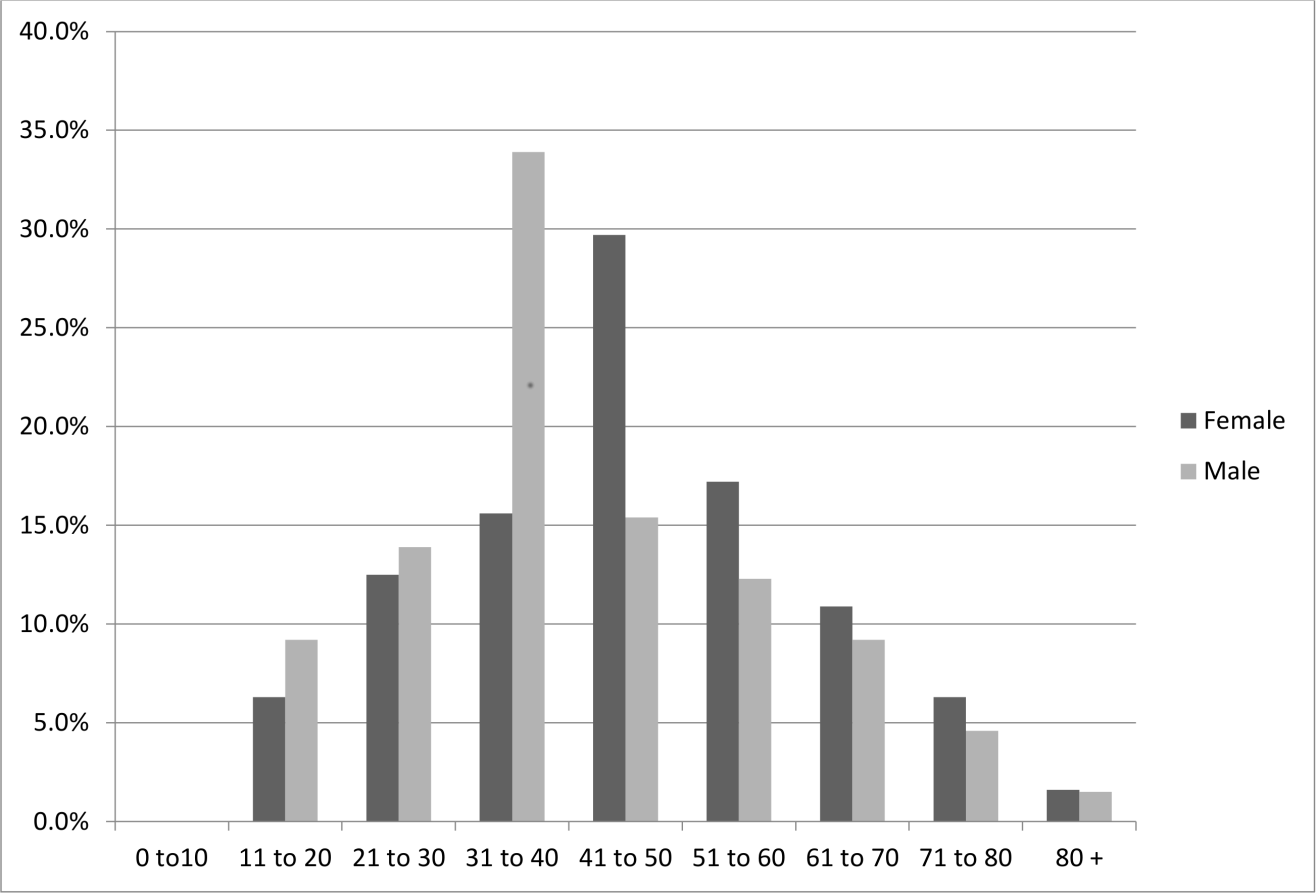
Incidence Rates of PGC by Gender and Age Groups.

The incidence rate for males was 32% compared to 23% for females (Table 1). There was a significant difference in the presence of PGC between males and females, χ^2^ (1, N = 480) = 4.74, *p* = 0.030. In addition, the incidence rates among the age groups were 22.7% for 11–20, 25.8% for 21–30, 37.2% for 31–40, 36.7% for 41–50, 38% for 51–60, 30.2% for 61–70, 26.9% for 71–80, and 16.7% for 80+ age group (Table 1), this was also statistically significant, χ^2^ (8, N = 480) = 40.22, *p* < .0001. The distribution of all patients according to the presence of PGC by age groups and gender are given in Fig 1. The highest incidence rate for males was the 31–40 year age group which had 33.9% PGC whereas the highest incidence rate for females was the 41–50 year age group, which had 29.7% PGC (Fig 1).

Table 2 shows the results of both the univariate and multivariate models. The logistic regression models demonstrated that age and gender were significant risk factors for pineal gland calcification. In the univariate model, males were 1.57 (95% CI, 1.04–2.36) times more likely to have pineal gland calcification compared to females. Similarly, for every year increase in age, the odds of an individual developing PGC increases by 1.02 (95% CI, 1.01–1.03) units. In the multivariate analysis, males were 1.94 (95% CI, 1.26–2.99) times more likely to have pineal gland calcification compared to females after controlling for the effects of age. For every one year increase in age, the odds of an individual developing PGC increases by 1.02 (95% CI, 1.01–1.03) units after controlling for the effects of gender.

**Table 2:** Logistic Regression assessing the relationship between age and gender, and PGC.

	Crude Odds Ratio (95% CI)	Adjusted Odds Ratio (95% CI)
Gender		
Female	1	1
Male	1.57 (1.04, 2.36)*	1.94 (1.26, 2.99)*
Age (in years)	1.02 (1.01, 1.03)**	1.02 (1.01, 1.03)**

*Note*:

*p< 0.05

**p<0.0001

## Discussion

The incidence of pineal gland calcification reported in the literature has varied depending on the quality of radiographic examination, the use of computed tomography versus skull radiography, the age of the population studied, and the ethnic and geographic makeup of the population. In our analysis of pineal gland calcification, we ascertained an incidence of 26.9% among 480 subjects between the ages of 3 and 89, utilizing skull x-rays. Compared to our analysis, lower incidence estimates have been reported by some studies [12,15,31] while higher incidence rates have been reported by others [32–34], suggesting variability in PGC incidence by geographic and ethnic makeup. In general, our study findings are consistent with the literature that PGC is more common in males than females and that it increases in incidence with aging.

The youngest patient with PGC in our study was a 15 year old male. This finding is consistent with previous studies, which indicated the occurrence of PGC as early as the second decade of life, and confirm the rarity of this condition in children younger than 10 years of age [31,35,36]. Thereafter, the incidence increases with age up to the sixth decade of life then it starts to decline; indicating that it is an age related process, which is consistent with previous studies [12,13,17,31,36]. The pattern of pineal calcification across ages in this study is that males showed more calcifications till 40 years while females showed more calcifications after 40 years.

Our finding regarding the preponderance of PGC among males is also consistent with previous reports that PGC is more common in males [11,12,32,34,37,38]. In contrast, a few studies using skull x-rays have found a slightly higher incidence among females [11,15]. Adeloye and Felson [12] found an equal sex distribution in the incidence of PGC in their analyses of 952 skull x-rays in Nigeria. Although the underlying mechanism behind this gender difference has not clearly been explained, most authors link this to the relationship between melatonin and sex hormones. Sandyk et al. [39] found that melatonin antagonizes the effect of estrogen by stimulating the production of progesterone. Additionally, Silman et al. [40] found that there is an abrupt reduction in melatonin levels among boys just before a rise in testosterone levels with advancing development, indicating its anti-gonadotropic effect. The above two findings led many authors to accept the low incidence of PGC in premenopausal women compared to men.

There is growing interest in the application of principles of evidence-based approaches to clinical radiology, which contributes to the strength of scientific evidence in the field of many medical disciplines. There is increased interest in the prevention of aging-related diseases. It has been reported that a number of the neurodegenerative diseases such as Alzheimer’s disease, Parkinson’s disease, depression, stroke, schizophrenia, multiple sclerosis, and sleep disorders may be associated with increased rate of PGC [16,18–24,26,27,41]. Therefore, studies that ascertain the incidence of age-related PGC in a region lacking such knowledge may have special importance in understanding various diseases in that region.

The main strength of our study is that it is the first to provide baseline information about the incidence of PGC in the Kurdistan Region of Iraq, but it is not without limitations. The incidence of PGC using skull x-rays could be slightly underestimated because plain skull x-rays have decreased sensitivity for identifying pineal calcification compared to computed tomography (CT) [35]. Therefore, we cannot rule out the presence of minute foci of calcification in the pineal gland. In addition, it is sometimes difficult to distinguish between pineal gland calcification and calcification in the immediate surrounding structures such as the choroid plexus and habenular commissure, which are considered extra-pineal calcifications. However, the majority of studies evaluating incidence and prevalence of PGC have used skull radiography because it is cost-effective, easily accessible, and frequently used in all clinical settings [11,14,15,38,42,43]. In addition, the generalizability of the findings in this study may be limited to people living in the Duhok Governorate and the other two governorates forming the Kurdistan Region. This is because people living in the middle and south of Iraq have a different ethnic background, and a different climate; therefore they may have different propensity for PGC than those of the Kurdish people. Finally, there may be selection bias in our sample. Only patients that sought care at the teaching hospital used for the study were included in the study. It is possible that there are other patients that sought care in private clinics and those individuals might have different characteristics from those that sought care at the study hospital. However, the study hospital is the largest and the main public hospital in Duhok Governorate and majority of people in the region seek care there, thus minimizing the effect of selection bias.

In conclusion, this study demonstrates a relationship between PGC development and age and gender in a Kurdish population sample. The findings of this study can serve as a preliminary data for future researchers to further investigate the nature and mechanisms underlying pineal gland calcification. Future researchers should explore possible associations between PGC and various disease presentations through collaborative efforts between radiologists and primary care physicians. In addition, future research should replicate this study using a large heterogeneous sample for greater generalizability, and should examine variables other than age and gender that correlates with PGC such as climate. Finally, future studies should focus on the relationship between melatonin and sex hormones, and their effects on PGC.

## Supporting Information

S1 Dataset_Analytic Analytical dataset used for analysis. (SAS7BDAT)

S1 Dataset_Original Original dataset used for analysis. (SAS7BDAT)
